# Vineyard under-vine floor management alters soil microbial composition, while the fruit microbiome shows no corresponding shifts

**DOI:** 10.1038/s41598-018-29346-1

**Published:** 2018-07-23

**Authors:** Ming-Yi Chou, Justine Vanden Heuvel, Terrence H. Bell, Kevin Panke-Buisse, Jenny Kao-Kniffin

**Affiliations:** 1 School of Integrative Plant Science, Cornell University, Ithaca, NY 14850 USA; 2 New York State Agricultural Experiment Station, Geneva, NY 14456 USA; 3 Department of Plant Pathology and Environmental Microbiology, Pennsylvania State University, University Park, PA 16802 USA; 4 United States Department of Agriculture, Agricultural Research Service (USDA-ARS), Madison, WI 53706 USA

## Abstract

The microbiome of a vineyard may play a critical role in fruit development, and consequently, may impact quality properties of grape and wine. Vineyard management approaches that have directly manipulated the microbiome of grape clusters have been studied, but little is known about how vineyard management practices that impact the soil microbial pool can influence this dynamic. We examined three under-vine soil management practices: 1) herbicide application, 2) soil cultivation (vegetation removal), and 3) natural vegetation (no vegetation removal) in a Riesling vineyard in New York over a three-year period. The microbiomes associated with soil and grapes were profiled using high-throughput sequencing of the bacterial 16 S rRNA gene and fungal ITS regions. Our results showed that soil bacterial composition under natural vegetation differs from that seen in glyphosate-maintained bare soil. Soil fungal composition under the natural vegetation treatment was distinct from other treatments. Although our study revealed soil microbiome shifts based on under-vine management, there were no corresponding changes in fruit-associated microbial composition. These results suggested that other vineyard management practices or environmental factors are more influential in shaping the grape-associated microbiome.

## Introduction

Vineyard management practices impact fruit and wine composition through many routes^1^. Widely known effects include the modification of the leaf area to fruit ratio^2^, alteration of the fruit microclimate^3^, and changes in nutrient and/or water uptake. However, the role of vineyard microbiology has been largely overlooked until recently, with researchers suggesting microbial composition as a possible driver of wine sensory properties^4^.

Aside from intentional inoculation, the major sources of yeasts in wine fermentations are derived from the vineyard and winery^5,6^. The impact of the winery environment on yeast dynamics during fermentation has been extensively studied^5,7–9^, but vineyard factors have received less attention. Recent studies have found that the microbial assemblages present in grape must and wine fermentation are structured to reflect regional and vineyard site patterns^10^, which suggests the importance of vineyard factors.

Grape microbiomes can be shaped by climate, region, site, and grape cultivar^11–14^, and also appear to be associated with the composition of the microbiome involved in wine fermentation, and with wine metabolite profiles and abundances^10^. Regionally-differentiated yeast genotypes collected from vineyards, forests, and spontaneous fermentations are confirmed to have different impacts on wine chemical composition^15^. These studies suggest the importance of a biological component to regional wine typicity through vineyard microbiome by indicating the significance of specific vineyard properties on wine characteristics as a function of microbiome composition. To express this role, “microbial terroir” was defined as traits of the land that impart a distinct profile of wine that is specific to the growing region, which suggests that the collection of bacteria and fungi from a region could contribute to regional wine characteristics^4,16^.

Management practices in the vineyard and winery, such as the use of fungal sprays and sulfiting fermentations, play important roles in the microbial dynamics of grape and wine fermentations that potentially contribute to wine sensory typicity^17^. Yeast populations in vineyard soil^18^, grapes, and wine fermentations^19–22^ have been studied in the context of overall vineyard management approaches, such as organic, conventional, and biodynamical management, where the direct manipulation of grape-associated microbiomes were involved. Differentiated grape microbial management using different phytosanitation sprayers derived predictable results, while masking the effect of other management practices on grape microbial ecology.

Others have investigated vineyard management practices that manipulated the soil environment, which is possibly the vineyard microbial pool^23^, without direct grape microbial manipulation. A study that was conducted in a hot and arid climate in Spain suggested that soil tillage is related to high diversity in grape-associated yeast^24^. However, they were unable to statistically test this concept as they relied on culture-dependent techniques to characterize yeast diversity. Another study conducted in California (USA) used high-throughput sequencing methods to demonstrate that vineyard floor management impacted the composition of soil bacteria^25^. Fungi were not included in their study, nor was the association of soil and grape microbial composition. Thus, whether vineyard management practices that change the vineyard microbiome can impact the grape-associated microbiome remains unclear.

The concept of soil as a source of microorganisms inhabiting grape surfaces is easily understood, but challenging to examine systematically. A study conducted in Long Island, NY (USA) found that bacterial communities associated with grape leaves, flowers, and fruit shared a greater proportion of taxa found in soil compared with each other, which they suggested as evidence of soil serving as a bacterial reservoir in vineyards^23^. There are several known microbial dispersal mechanisms that transport fungi and bacteria from the ground to crops, including rain^26^ and wind^27^. These routes of microbial dispersal are likely to hold in vineyards as well, although there are many other possible routes to be explored. Thus, it is possible that vineyard soil management practices could alter the microbiome in the vineyard - not only at the soil level, but also with aerial parts such as grapes. In one of our previous studies conducted in New York^28^, we showed that under-vine soil treatments had no impact on vine growth and yield components, but that wine sensory properties differed. We suspected the differences in wine sensory properties might have been a result of changes to the grape associated microbiome that altered secondary metabolite production in grapes or yeast dynamics in the wine fermentation. Under-vine floor management practices might have triggered changes in the grape associated microbiome.

To understand the vineyard management impacts on grape-associated microbiomes, a three-year single-factor (under-vine soil management) study was conducted within an experimental design that corresponds to our previous study^28^ in a commercial vineyard in the Finger Lakes region of New York. Under-vine soil management was chosen as our vineyard management factor, as we expected that it would directly manipulate the vineyard microbial pool in soil. The objective was to examine how under-vine soil management practices, including herbicide application with glyphosate (GLY), soil cultivation (CULT) using hand weeding, and under-vine natural vegetation (NV) with no cultivation or herbicides, impacted the microbiomes of soil and grapes. The goal of the study is to understand the role of specific vineyard soil management practices on the grape-associated microbiome. We hypothesized that under-vine soil management practices resulting in different types of ground vegetation, through glyphosate application, soil cultivation, or maintaining intact natural vegetation, would result in distinctly different soil and fruit microbial communities.

## Results

### Fungal communities cluster distinctly between soil and grapes

**Figure 1:**
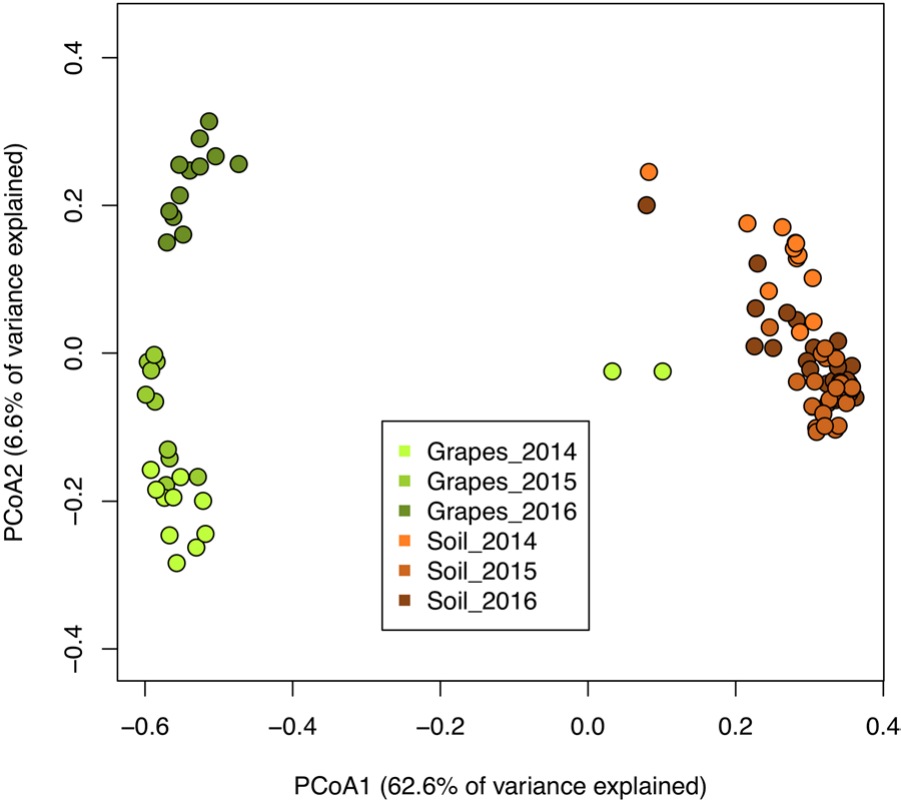
Principal coordinates analysis (PCoA) of fungal communities (ITS region) of soil, and grape from all harvest years and management treatments. The ordination is based on the Bray-Curtis distance metric, with samples clustering by collection type (grape and soil).

Fungal community profiles showed distinct clustering of samples derived from grapes, and soil collected under grapevines (Fig. 1). The Bray-Curtis distance metric was used to determine multivariate sample distances, which were visualized through an ordination of a principal coordinates analysis (PCoA). Axes 1 and 2 explained 69% of the variance in the data. The soil samples clustered together distinctly, and separately from grapes along the first PCoA axis, which explained 66% of the variance in the data (Fig. 1). Shannon diversity indices for OTUs differ between soil and grape samples (Supplementary Fig. S1), but no diversity differences were observed among treatments within the sample type in each year.

### Under-vine soil management impacted soil fungal community structure

**Figure 2:**
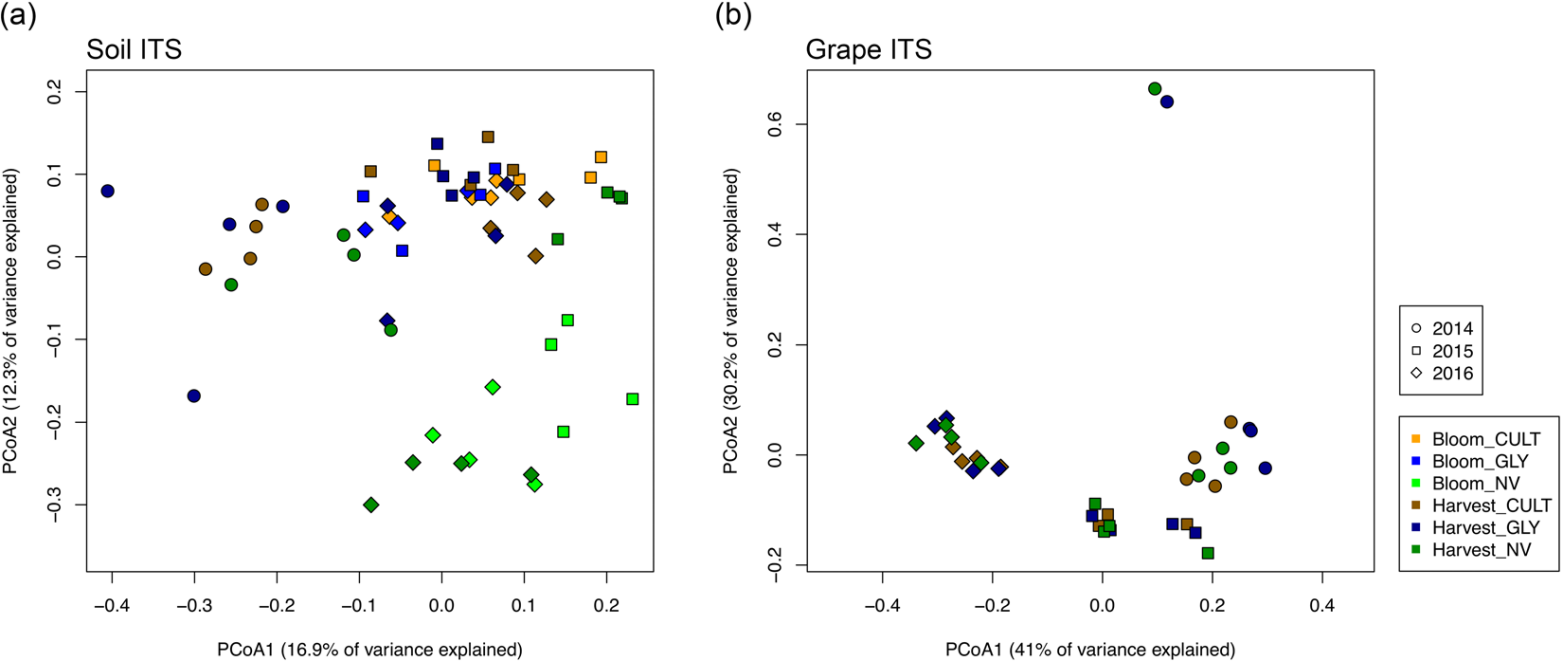
Principal coordinates analysis (PCoA) ordinations of fungal communities (ITS region) derived from (**a**) soil at grapevine bloom and harvest; and (**b**) grape at harvest. The three under-vine management treatments include Cultivation (CULT), Glyphosate (GLY) and Natural Vegetation (NV). The PCoA is based on the Bray-Curtis distance metric for three experimental years.

We evaluated the impact of under-vine soil management on microbial community composition. The three-year average under-vine soil vegetation coverage rate for NV was greater than 70%, while coverage rates for CULT and GLY were less than 20% at veraison. PCoA plots with samples from each of the three years of the study (generated using the Bray-Curtis distance metric) showed that NV soil fungal communities differed from those of GLY and CULT treatments (Fig. 2a). Over the three years of the experiment, sample clustering was based primarily on vintage, with each vintage clustered, and then by treatment, where NV separated from GLY and CULT. However, no clustering pattern was detected among the CULT and GLY samples. Notably, the dissimilarities between NV and the other two soil treatments grew with time since groundcover establishment, suggesting possible intensification of the NV treatment effect over time. In 2015 and 2016, the soil samples were taken at two different vine phenological stages - bloom and harvest, which showed separation by PCoA ordination.

These observations were confirmed by statistical analysis. According to the three-year overall Permutational Multivariate Analysis of Variance (PERMANOVA), vintage and treatment effects were both significant (P < 0.001), while year-to-year climatic differences (R^2^ = 0.159) explained more variation than treatment (R^2^ = 0.114). The treatment effect was significant across all three years (p = 0.032 in 2014, p = 0.001 in 2015 and p = 0.001 in 2016) when each year was analyzed individually. The phenological stage effect was significant in both year 2015 (p = 0.008) and 2016 (p = 0.048), while samples were not taken at vine full bloom in 2014 (Table 1).

**Table 1:** Comparison of bacterial and fungal community structure dissimilarity in soil and grapes using permutational multivariate analysis of variance (PERMANOVA).

Factors	Overall		2014		2015		2016	
	R^2^	P-value	R^2^	P-value	R^2^	P-value	R^2^	P-value
**Soil 16** **S**								
Dispersion test	—	—	—	0.322	—	0.571	—	0.044
Treatment	—	—	0.243	0.042	0.097	0.181	0.104	0.013
Stage	—	—	—	—	0.061	0.032	0.094	< 0.001
Treatment*Stage	—	—	—	—	0.083	0.757	0.085	0.176
**Soil ITS**								
Dispersion test	—	—	—	0.374	—	0.254	—	0.181
Treatment	0.114	<0.001	0.246	0.032	0.213	<0.001	0.243	<0.001
Stage	0.012	0.443	—	—	0.074	0.008	0.054	0.048
Year	0.159	<0.001	—	—	—	—	—	—
Treatment*Stage	—	—	—	—	0.094	0.066	0.058	0.653
Treatment*Year	0.082	<0.001	—	—	—	—	—	—
**Grape ITS**								
Dispersion test	—	—	—	0.690	—	0.747	—	0.765
Treatment	0.026	0.658	0.138	0.492	0.211	0.472	0.169	0.278
Year	0.498	<0.001	—	—	—	—	—	—
Treatment*Year	0.051	0.771	—	—	—	—	—	—

The p-values of dispersion test were derived from ANOVA.

**Figure 3:**
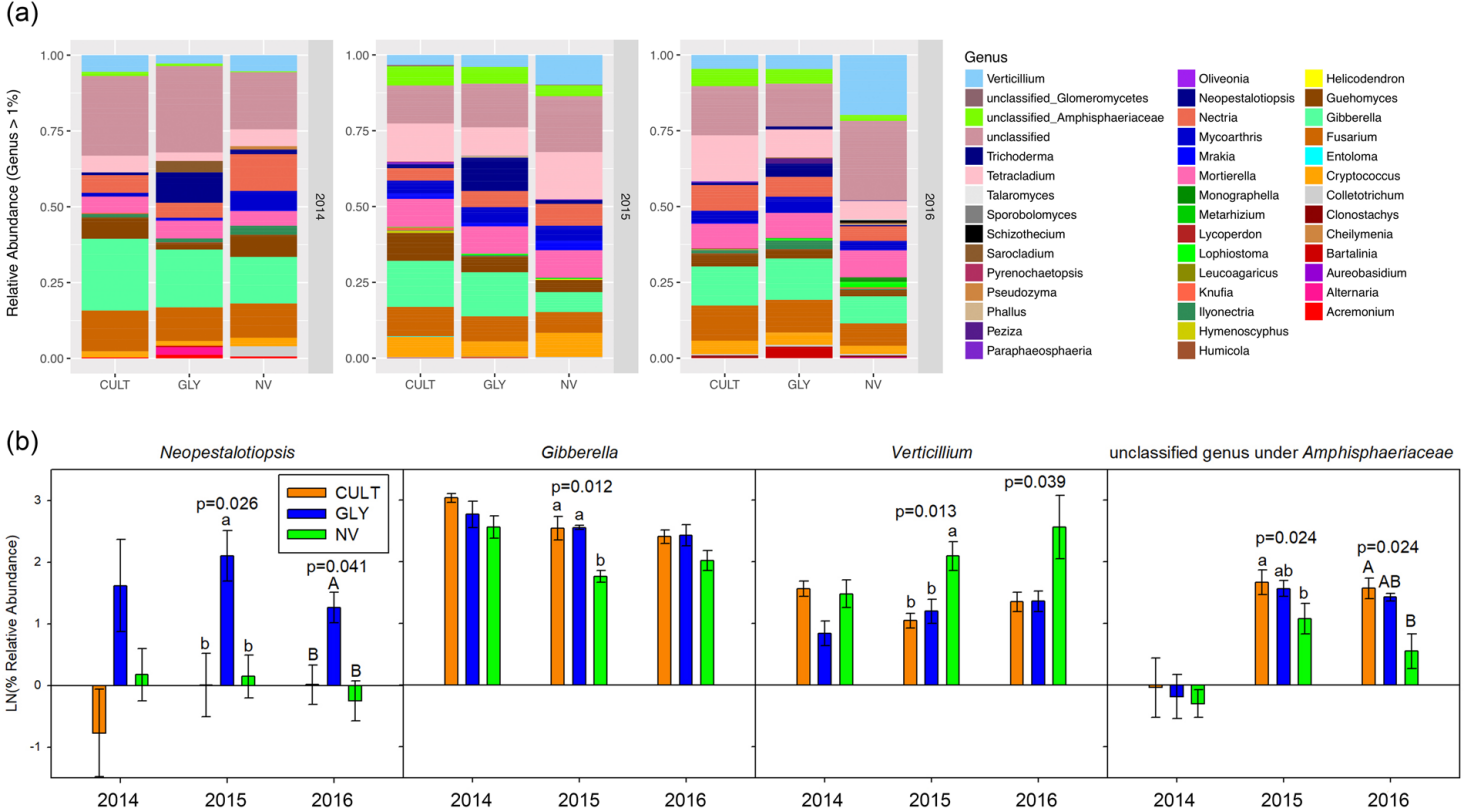
Mean fungal relative abundance at genus level. (**a**) Full fungi profile (>1%) in the soil from Cultivation (CULT), Glyphosate (GLY) and Natural vegetation (NV) field treatments (n = 4) and (**b**) Selective fungi that were different in relative abundance (n = 4) with standard errors. The statistical differences were tested by using one-way analysis of variance (ANOVA) followed with Tukey HSD test comparing log mean relative abundance at α = 0.05. The p-values were derived from ANOVA.

Unclassified fungal genera in soil samples ranged from around 10% to more than 25% relative abundance. However, analyses excluding the unidentified genera did not change the differentiation of NV samples from CULT and GLY samples on the ordination. The top five fungal genera found in the soil (excluding unclassified) were *Verticillium*, *Nectria*, *Mortierella*, *Gibberella* and *Fusarium*, based on average relative abundances across all soil samples (Fig. 3a). Fungal genera relative abundance differences were found in *Gibberella*, *Neopestalotiopsis*, *Verticillium* and an unclassified genus under *Amphisphaeriaceae* family, where NV soils contained fewer *Gibberella* (P < 0.005 in 2015) and more *Verticillium* (P < 0.05 in 2015 and 2016) compared to the other two treatments, and less *Neopestalotiopsis* (P < 0.05 in 2015 and 2016) and unclassified *Amphisphaeriaceae* (P < 0.05 in 2015 and 2016) relative to GLY soils. CULT soils had less *Neopestalotiopsis* (P < 0.05 in 2015 and 2016) compared to GLY soils (Fig. 3b). Among these genera, *Neopestalotiopsis* and *Verticillium* are found in the top five most important variables along with *Monographella*, *Paraphaeosphaeria* and unclassified genera under *Nectriaceae* in the Random Forest model for soil treatment prediction (Supplementary Fig. S2).

### Under-vine soil bacterial community structure was impacted by floor management practice

**Figure 4:**
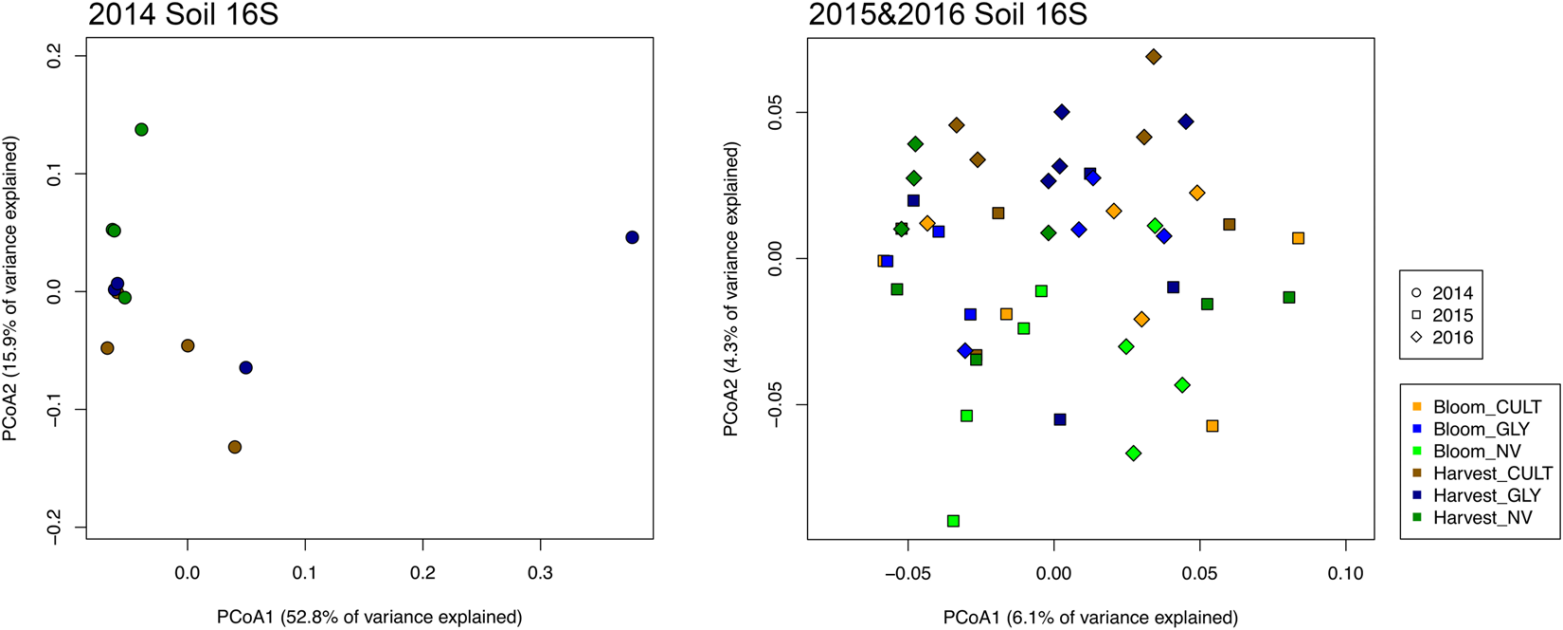
Principal coordinates analysis (PCoA) ordinations of soil sample bacterial microbiota derived from Cultivation (CULT), Glyphosate (GLY) and Natural vegetation (NV) field treatments at bloom (B) and harvest(H) based on weighted UniFrac distance metric for 2014, 2015 and 2016 experimental years, where year 2014 was analyzed apart from 2015 and 2016 due to the amount of sequences difference in the samples.

The sequencing reads generated from the 2014 samples contained unexpectedly high amounts of short reads, whereas the sample sequences were comparatively low. Although the quality of the remaining reads was sufficient for a within-year comparison, we decided to present the data year-by-year instead of the three-year overall analysis, due to the dramatic difference in read depth relative to 2015 and 2016 samples. Although the samples did not seem to cluster based on treatments on PCoA plots using UniFrac distance metrics (Fig. 4), the treatment effect was significant in year 2014 (p = 0.042) and 2016 (p = 0.013) according to PERMANOVA (Table 1). In fact, paired-PERMANOVA further revealed that the bacterial community structure among the treatments was different in 2014, where NV differed from GLY (p = 0.026) and CULT (p = 0.033), and 2016, where NV differed from GLY (P = 0.036) (Table 2). Grape-associated bacterial community structure was not further examined due to low yield of bacterial DNA resulting in low PCR amplification.

**Table 2:** Comparison of bacterial and fungal community structure dissimilarity in soil and grapes using paired-PERMANOVA.

Pairs	overall		2014		2015		2016	
	R^2^	p value	R^2^	p value	R^2^	p value	R^2^	p value
**Soil 16** **S**								
CULT vs GLY	—	—	0.135	0.621	0.070	0.481	0.064	0.455
CULT vs NV	—	—	0.186	0.033	0.072	0.209	0.088	0.064
GLY vs NV	—	—	0.190	0.026	0.083	0.086	0.088	0.036
**Soil ITS**								
CULT vs GLY	0.061	0.005	0.157	0.238	0.110	0.027	0.103	0.022
CULT vs NV	0.101	<0.001	0.225	0.095	0.179	0.001	0.251	0.002
GLY vs NV	0.097	<0.001	0.208	0.105	0.205	<0.001	0.197	<0.001
**Grape ITS**								
CULT vs GLY	0.022	0.860	0.186	0.103	0.089	0.809	0.135	0.608
CULT vs NV	0.021	0.894	0.113	0.912	0.172	0.421	0.155	0.514
GLY vs NV	0.017	0.834	0.051	0.581	0.205	0.206	0.105	0.835

### Under-vine soil management did not impact fungal communities on grapes

Grape samples were collected at commercial harvest in each year. Over 71% of the variance in grape fungal community structure was explained by the first two PCoA axes, but the grape samples were not structured as a function of under-vine soil treatments (Fig. 2b). PERMANOVA and paired PERMANOVA were used to confirm that no community composition differences were found among treatments. The three-year overall PERMANOVA showed that the year-to-year differences were the only significant effects (Table 1).

Unclassified genera accounted for 5 to more than 30% of the relative abundance in grape samples. The top five fungal genera with the highest average relative abundance of the three years in the grape samples were *Sporobolomyces*, *Aureobasidium*, *Rhodosporidium*, *Penicillium*, and *Entyloma*. The fungal genera that differed in relative abundance in soil were not found to differ in relative abundance in grapes. Differences in relative abundance in grape-associated fungal genera were found in *Penicillium*, *Sporobolomyces* and unidentified genera across the years. The fungal genus *Penicillium* was found only in the 2014 grape samples, which was 16.6% in relative abundance, and *Sporobolomyces* was highest in relative abundance in 2015 (p < 0.05) and lowest in 2016 in grape samples (p < 0.01), and the unidentified genera relative abundance in 2016 was higher than that in 2014 and 2015 (p < 0.0001) (Supplementary Fig. S3). The differences in these fungal genera may account for the separation of the grape samples by vintage on the PCoA plot. The grape-specific (not found in soil) fungal genera detected included *Coprinellus*, *Ischnoderma*, *Mycosphaerella*, *Occultifur*, *Pestalotiopsis*, and *Tilletiopsis*. Many yeast genera commonly found in abundance in grapes, such as *Candida*, *Pichia*, *Debaryomyces*, *Lipomyces*, *Kluyveromyces*, and *Issatchenkia*, were not found or were not abundant (<1% in relative abundance) in this study.

## Discussion

The link between soil microbiome composition and regional wine characteristics has been recently studied^10,15^, leading to greater interest in the role of microbes in fruit and wine composition^4^. Our multi-year experiment examined whether different under-vine soil management practices could alter grape-associated microbial composition. While a previous study suggested that soil management in the vineyard can impact soil microbial assemblages^25^ and that grapevine aerial organ-associated bacterial OTUs likely originated from soil^23^, we hypothesized that implementing different under-vine soil management practices would not only alter soil microbial composition, but that the grape-associated microbiomes would reflect these changes. In our study, changes in the fungal community of the soil, due to adopting different under-vine soil management practices, did not extend to the grapes, leading us to reject our hypothesis for our study site.

Previous studies have shown that vineyard management alters grape and fermentation microbiome composition where systematic vineyard management practices or direct microbial management approaches were applied^17,19,20,29^. In one study, yeast dynamics during the spontaneous fermentation using grapes obtained from conventionally and non-conventionally managed vineyards differed^20^. Another study revealed that management practices applied directly onto grapes, such as pesticides, impacted grape-associated yeast diversity, which negatively correlated with the copper residuals found on the grapes^19^. Unlike these studies, our study did not directly manage the microbes on the grapes, but applied indirect changes to microbial composition in soils. Our study showed a link between under-vine management practices and soil bacterial and fungal composition, which confirmed results from previous studies that focused on soil bacterial^25^ and fungal composition^22^. The results of this study further revealed no corresponding changes in the grape fungal microbiome, which does not dispute findings from another study^22^. The researchers reported that the juice fungal microbiome obtained from conventionally and biodynamically managed vineyards did not differ from each other, despite showing that fungal populations on the grape surface differed by vineyard management approaches.

In our study, under-vine soil treatment impacts on grape fungal composition could also be masked by factors such as climate, geological properties (e.g. soil type), management practices associated with cool climate viticulture (e.g. trellis system, fungal spray use and frequency), vineyard management history, and inter-row vineyard floor management. Among these factors, many are specific to the region, such as large vine size with tall trellis systems, frequent pesticide applications, and hilling soil up over the graft union in winter and down off of the graft union in the spring. In a broader sense, climatic conditions play a significant role in microbiome structure, which is shown in our study, with year-to-year climate differences being the most significant factor explaining variance in soil and grape fungal assemblages, which is consistent with a previous study^11^.

With weather variability increasing as a function of climate change, there is renewed interest in improving resilience of vines to environmental stress. Cover crops are known to improve soil health by retaining soil moisture, enhancing drainage, raising soil organic matter content, maintaining soil physical structure, and supporting soil microbial properties and processes^30–34^. Also, cover crops provide a prolific root zone (rhizosphere) that enriches for a diversity of microorganisms that perform many functions, such as mediating soil nutrient cycling, impacting plant growth and development, and influencing pathogen interactions^30,31,35–37^. This may require long-term assessment, as no soil microbial diversity difference was observed between bare soil and vegetative soil over the course of three years. However, in our study, we did observe a lower relative abundance of *Neopestalotiopsis* and an unidentified genus under *Amphisphaeriaceae* in the soil with vegetation, which may possibly relate to grapevine trunk pathogenic species^38^. Since our study only examined short 16 S rRNA gene and ITS reads, we are not able to determine whether specific organisms we identify are pathogenic or not.

This study aimed to evaluate the role of management practices - specifically vineyard soil management - on the vineyard microbiome. We found that bare soil maintained by soil cultivation and herbicide led to soil bacterial and fungal communities that diverged from the non-cultivation natural vegetation treatment. The results indicate that vineyard microbiome could be susceptible to changes under different soil management practices; however, the spatial gap between soil and the fruiting zone, and the frequent pesticide applications, could impact the level of soil management effects. It also suggests that future studies on the movement of microorganisms from soil to grape would be key to understanding the role of vineyard soil management in shaping the microorganisms associated with grapes.

Despite previous findings on vineyard management effects on vineyard microbiomes, we show that altering soil microbial composition in the vineyard through under-vine management practices did not result in corresponding changes to the grape microbiome at our study site. The concept that soil microbial composition could be impacting fruit and wine composition should be examined in light of vineyard management practices that alter soil biotic components. Regional management practices such as ground cover management, height of the trellis system, and phytosanitation, that respectively modify soil conditions, transportation of microbes from soil to grapes, and grape-associated microbes, could have a significant role in shaping fruit and wine composition in vineyards.

## Methods

### Vineyard design

The experiment was conducted in a commercial vineyard on Howard gravelly loam soil located in Ovid, NY, USA for three consecutive years from 2014 to 2016. The vines, *V*. *vinifera* cultivar Riesling grafted onto 3309 C rootstock, were planted in 2001 with 2.13 m × 2.74 m intra- and inter-row spacing. The trellis system was cane pruned Scott-Henry system with 10 buds per cane on each of four canes. A complete randomized block design was applied to enable four replicates for each treatment, and the treatments were randomly assigned to the experimental units, which are one meter wide under-vine soil strips, within each block. Each experimental unit was across three rows with nine consecutive vines in a row. The grape and soil samples were collected from the middle three vines and the accordance under-vine 1 m × 5.8 m soil strip, in the middle row from each of the experimental unit where the other vines were served as guards for physical and spatial buffering. The vineyard canopy, pest-control and amendments were managed following standard commercial practice in the Finger Lakes region^39^ by the professional vineyard crew.

### Under-vine soil treatments

The experimental units were subjected to three different under-vine soil treatments in a one meter wide strip under vines including spot application of herbicide, in which the active ingredient was glyphosate, cultivation maintained bare soil, and natural vegetation, where weeds grew freely with periodic mowing to keep them out of the fruiting zone. Herbicide and cultivation bare soil strips were established following the commercial standard. In brief, 2% Roundup (Monsanto, MO, USA) was sprayed with electronic pumped spraying nozzle in rate about 3 kg a.i./ha. Cultivation was done by combining mechanical, rototiller to roughly 20 cm depth, and manual tillage, cultivation with hoes. Herbicide was applied on June 23^rd^, July 9^th^, July 18^th^ in 2014, June 16^th^ in 2015 and June 15^th^ in 2016. Soil cultivation was applied on June 27^th^, July 3^rd^, and July 18^th^ in 2014, June 3^rd^, July 23^rd^ to July 27^th^ in 2015 and May 25^th^ and June 24^th^ in 2016. A permanent between-row cover crop was maintained separately and was a mix of fescue, white clover and weeds.

### Sample collection

At bloom (2015 and 2016) and harvest (2014, 2015 and 2016), ten soil cores per experimental unit were collected using sterile cores (6 cm diameter × 10 cm deep) attached to the slide hammer auger (AMS Inc, American Falls, ID, USA) in a grid pattern. Grape cluster samples were taken at commercial harvest with individual sterilized blazers for each of the experimental unit. Ten clusters from each experimental unit were randomly picked. Sub-samples from each of the experimental units were combined in sterile containers in the field, transported on ice, and stored at −20 °C until further analysis. Sub-samples of five berries per cluster, comprised of two from the top, two from the middle and one from the bottom of the cluster, were detached in the original field sampling container while frozen, and allocated into a new container to make 50 berries per experimental unit for grape DNA extractions. The soil sample of each experimental unit was thawed at room temperature, fully homogenized, and then 0.25 g of soil was taken carefully, avoiding any non-soil particles for downstream DNA extraction.

### Sample DNA extraction, amplification and Sequencing

DNA extraction of soil samples followed the protocol for the PowerSoil DNA isolation kit (MO BIO Laboratories, CA, USA). For grape samples, the grapes were thawed and crushed in the zip bag before following the procedures. For each experimental unit, grape must sample was vortexed and homogenized, and transferred into two 2 ml Eppendorf tubes and centrifuged at 11600 × g for 20 minutes. The pellets from the same sample were combined and washed two times with chilled PBS. The pellets were then used for DNA extraction following the protocol from the MoBio PowerPlant DNA isolation kit. The bacterial 16 S rRNA gene V3/V4 regions and fungal ITS barcoded region were amplified with the universal bacterial primers 341 F (5′-CCTACGGGNGGCWGCAG-3′) and 805 R (5′-GACTACHVGGGTATCTAATCC-3′) and fungal primers ITS1F (5′-CTTGGTCATTTAGAGGAAGTAA-3′) and 5.8A2R (5′-CTGCGTTCTTCATCGAT-3′), in which the Illumina adaptors at the 5′ end of the primer sequences (5′-TCGTCGGCAGCGTCAGATGTGTATAAGAGACAG-3′ for the forward primer and 5′-GTCTCGTGGGCTCGGAGATGTGTATAAGAGACAG-3′ for the reverse primer) were attached^40,41^. The reaction was conducted in 20 μl containing 9 μl H_2_O, 8 μl 5prime HotMaster mix (5 PRIME Inc., MD, USA), 1 μl of each primer (forward and reverse) and 1 μl of 1:10 diluted DNA template in thermocycler (Bio-Rad, CA, USA) following the condition of 3 min at 95 °C and then 25 (bacteria) and 30 (fungi) cycles of 30 s at 95 °C, 35 s at 50 °C and 60 s at 72 °C before entering the final step of 10 min at 72 °C. The amplicons were transferred into 96-well plates and cleaned with MagBio HighPrep PCR beads (MagBio Genomics, MD, USA). We then attached unique two-barcode indexes to cleaned amplicons by running PCR with 2.5 μl each of forward and reverse primers (10 µM) carrying designated barcodes, 12.5 μL of Q5 High Fidelity 2× Master Mix (New England Biolabs Inc., MA, USA), 5 μL of template, and 2,5 μl of water, with the following temperature protocol: 8 cycles of 15 s at 98 °C, 30 s at 55 °C and 20 s at 72 °C after 1 min at 98 °C and before 3 min at 72 °C. Sample DNA was normalized with the SequalPrep Normalization Kit (ThermoFisher, Waltham, MA), pooled using equal liquid volumes, and the pool purified with a PureLink QuickGel Extraction Kit (ThermoFisher). Each pool was sent to the Cornell Institute of Biotechnology (Ithaca, NY) for paired-end sequencing, using the 600-cycle MiSeq Reagent Kit v.3 for our 16 S pool, and the 500-cycle MiSeq Reagent Kit v.2 for our ITS pool on the Illumina MiSeq platform (Illumina Inc., CA, USA). The sequencing process generated 4,060,310 ITS and 552,871 16 S rRNA gene reads after downstream processing as described below. All the MiSeq data were uploaded to the NCBI Sequence Read Archive and are public accessible under the project number of SRP132177.

### Bioinformatic and statistical analysis

The raw sequences were processed and aligned following the protocol described in the Brazilian Microbiome Project^42^ with some modifications^43^. Briefly, paired-end sequence merging, primer trimming, and singleton sequence removal were performed in Mothur v 1.36.1. Operational Taxonomic Units (OTU) were produced at 97% sequence similarity. Taxonomic classification of OTUs was performed in Mothur using the GreenGenes v.13.8 database for 16 S rRNA gene sequences and UNITE v. 7 database for ITS sequences. Suspected non-bacterial and non-fungal OTUs, including chlorophyll and mitochondria, were also removed in Mothur. All downstream data analysis was conducted in R version 3.3.3 with packages Vegan and Phyloseq. The microbial diversity was determined using Shannon Diversity Index using “diversity” function in package vegan. The β-diversity of the assemblage dissimilarity between samples were calculated with the Bray-Curtis distances for fungal community and weighted UniFrac distances for the bacterial community using package vegan. The dissimilarity matrices obtained were also used for Principal Coordinate Analysis (PCoA) plotting against the first two dimensions (highest variables explained). Multivariate dispersion analysis was performed to test the differences in variances among the treatments using command “betadisper” in package vegan where the β-diversities were obtained based on the Bray-Curtis distance metric for fungal community and UniFrac distances for the bacterial community. Permutational Multivariate Analysis of Variance (PERMANOVA) and paired PERMANOVA using “adonis” command in package vegan at 999 permutations and α = 0.05 were performed testing factors including year (for overall analysis only), stage (for soil samples in 2015 and 2016 only), and under-vine soil treatments. When three-year overall analysis was conducted, the year was positioned as a fixed effect with samples within each block in constrained permutation to account for the repeated measures. The overall PERMANOVA was not performed for soil 16 S data due to large differences in sequencing depth between 2014 and the other years (average 450 reads per sample in 2014, and 11648 reads per sample in 2015 and 2016). Paired-PERMANOVA was performed by subsetting the treatments and applying Bonferroni correction to the P-values. The relative abundance of selected fungal genera in the samples were compared using one-way analysis of variance (ANOVA) test followed by Tukey HSD performing in JMP Pro 12.0.1 (SAS Institute, NC, USA), with log transformations when needed under violations of normality.

### Data availability

All of the data are provided fully in the result section within and supplementary data accompanying this paper.

## Electronic supplementary material

Supplementary Information
