# Supplementary material for: Vineyard under-vine floor management alters soil microbial composition, while the fruit microbiome shows no corresponding shifts
Source: Sci Rep. 2018 Jul 23;8:11039. doi: 10.1038/s41598-018-29346-1 (PMC6056419; doi:10.1038/s41598-018-29346-1)
Supplement: Supplementary file 1 — Supplementary Information [file 41598_2018_29346_MOESM1_ESM.docx]

**Vineyard under-vine floor management alters soil microbial composition, while the fruit microbiome shows no corresponding shifts**

**Ming-Yi Chou^1,2^, Justine Vanden Heuvel^1,2^, Terrence H. Bell^3^, Kevin Panke-Buisse^4^ and Jenny Kao-Kniffin^1^***

^1^ School of Integrative Plant Science, Cornell University, Ithaca, NY, 14850, USA

^2^ New York State Agricultural Experiment Station, Geneva, NY, 14456, USA

^3^ Department of Plant Pathology and Environmental Microbiology, Pennsylvania State University, University Park, PA, 16802, USA

^4^ United States Department of Agriculture, Agricultural Research Service (USDA-ARS), Madison, WI, 53706, USA

*Corresponding Author:

J. Kao-Kniffin

135 Plant Science Building

Cornell University

Ithaca, NY 14853, USA

Phone: (607) 255-8886

Fax: (607) 255-0599

E-mail: [jtk57@cornell.edu](mailto:jtk57@cornell.edu)

**Running Title: Vineyard management and microbiomes**


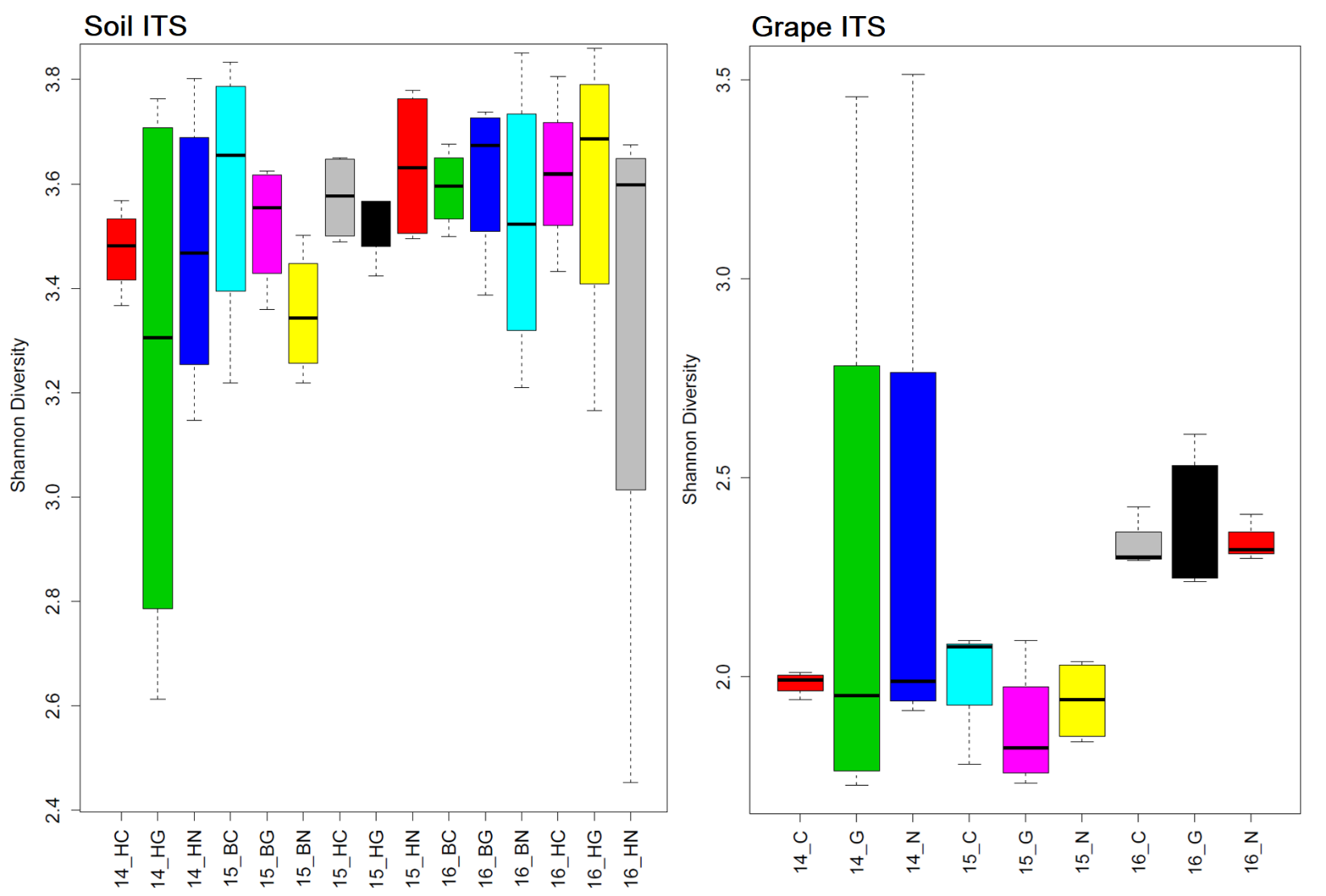


Figure S1. Fungal diversity in the under-vine soil at two different vine developmental stages, bloom and harvest, and the accordance grapes at harvest from three experimental years (2014-2016) analyzed using Shannon Diversity Index. In the x-axis, the numbers in the labels indicate vintage, H/B indicates the sampling stage at harvest/bloom, and the C/G/G indicates the soil treatments CULT/GLY/NV.


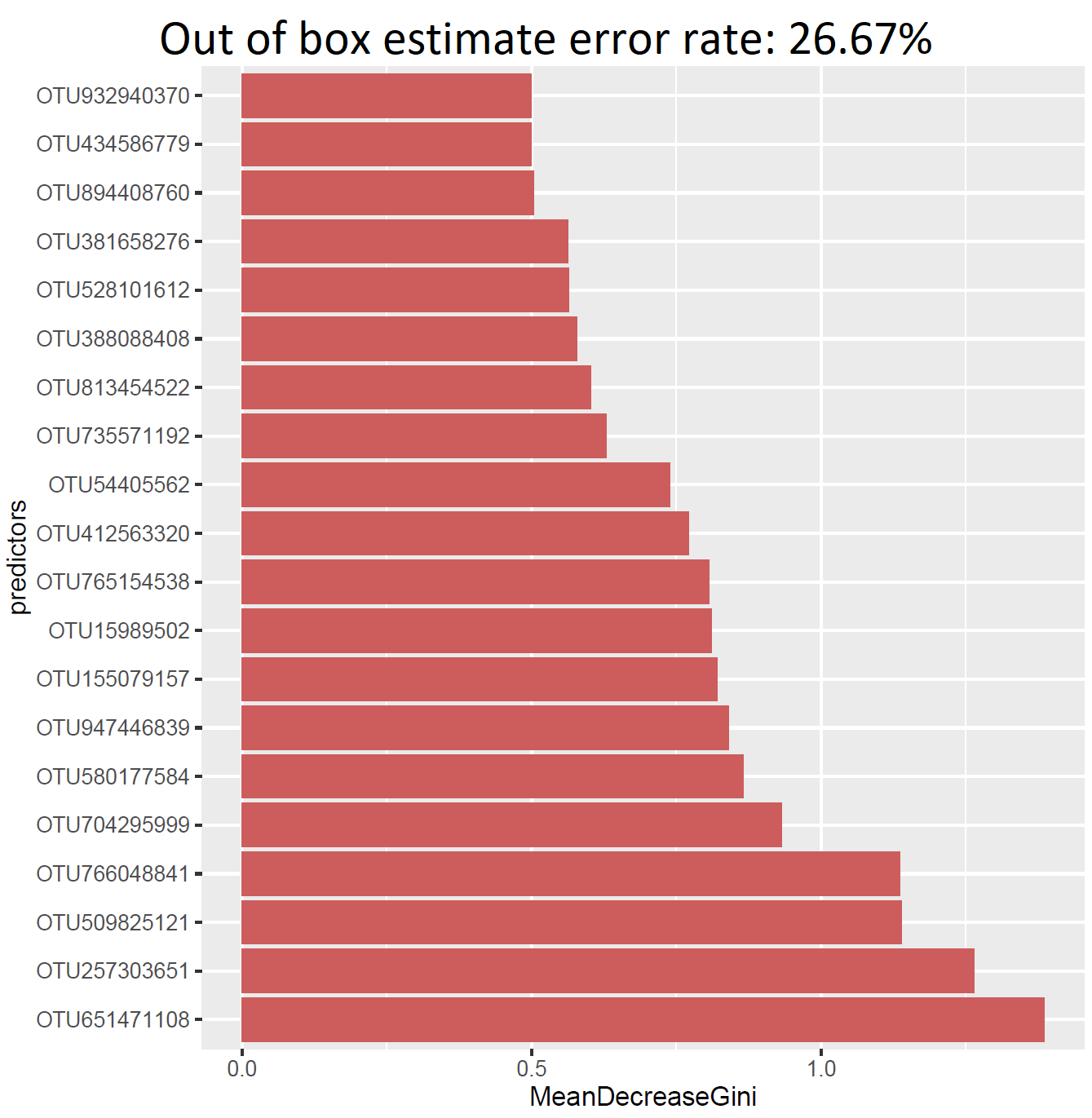


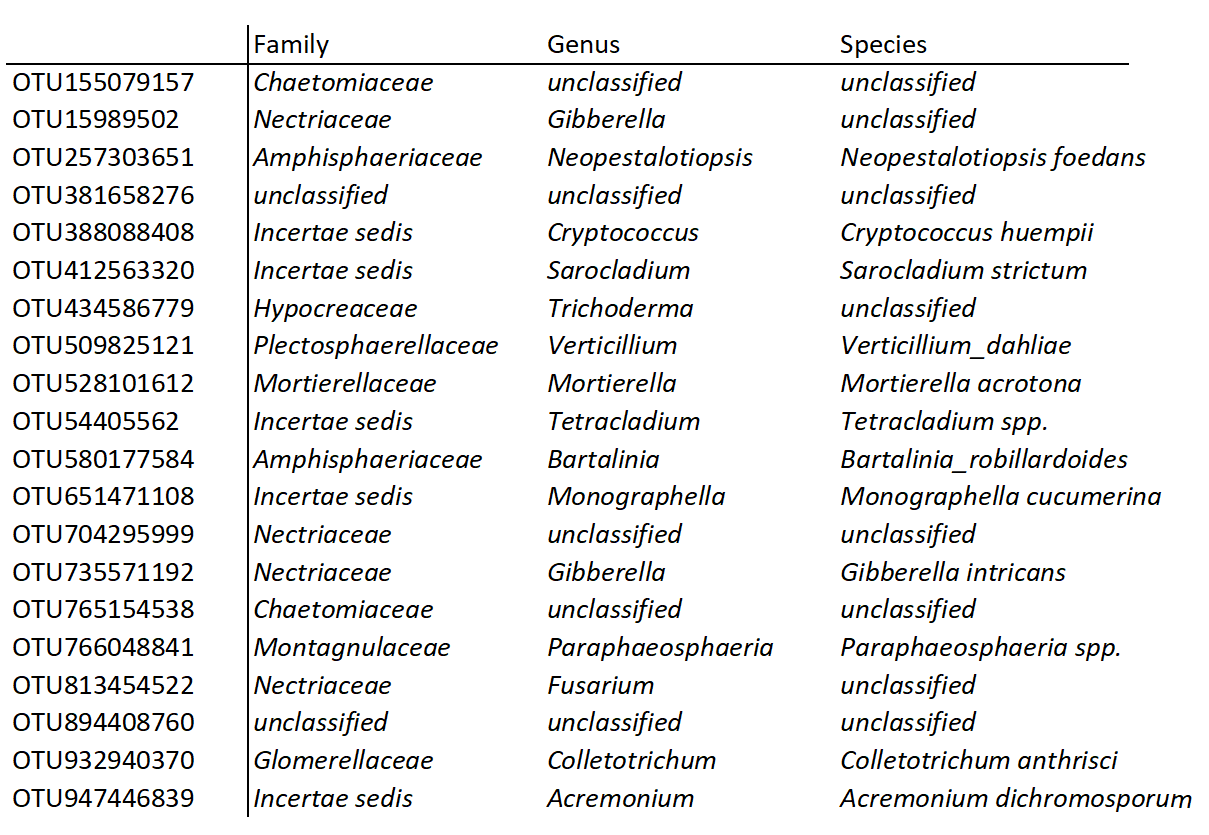


Figure S2. Under-vine soil treatment prediction derived from Random Forest model using soil fungal OTUs as variables. List of top 20 most important predictors and their correspondent fungal taxonomy according to their mean decrease in Gini coefficients.


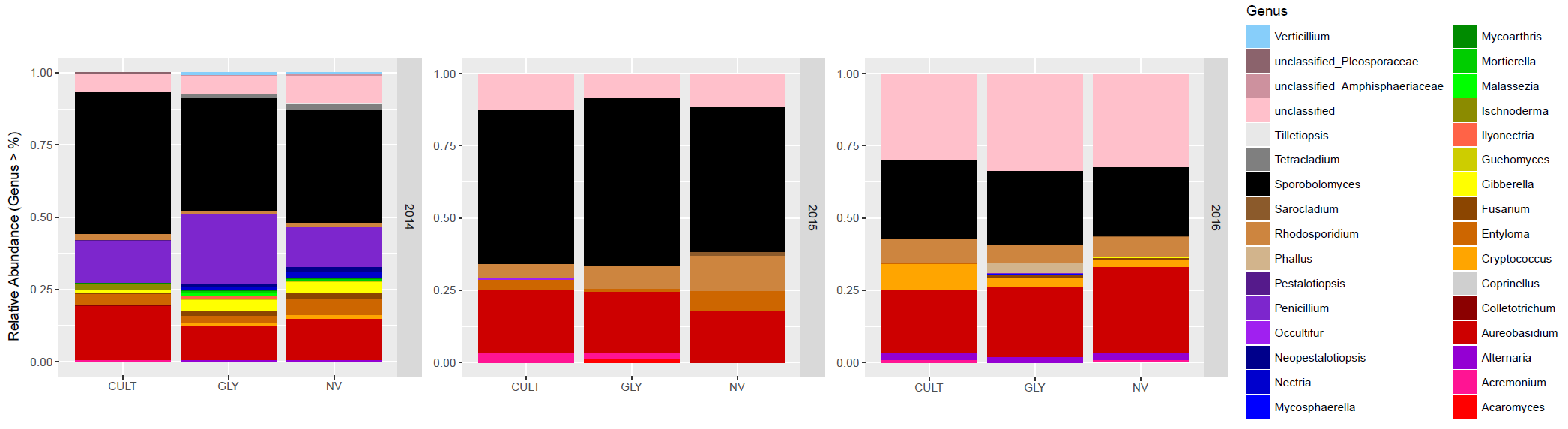


Figure S3. Fungal relative abundance of grape samples from Cultivation (CULT), Glyphosate (GLY) and Natural vegetation (NV) field treatments at genus level for three consecutive experimental years. Only the fungi genera with more than 1% mean relative abundance of all the replications within each treatment were presented.
